# HHcy Induces Pyroptosis and Atherosclerosis via the Lipid Raft-Mediated NOX-ROS-NLRP3 Inflammasome Pathway in apoE^−/−^ Mice

**DOI:** 10.3390/cells11152438

**Published:** 2022-08-06

**Authors:** Sijun Liu, Jun Tao, Fengqi Duan, Huangjing Li, Hongmei Tan

**Affiliations:** 1 Department of Pathophysiology, School of Medicine, Sun Yat-sen University, Shenzhen 518107, China; 2 Department of Pathophysiology, Zhongshan School of Medicine, Sun Yat-sen University, Guangzhou 510080, China; 3 Laboratory Animal Center, Sun Yat-sen University, Guangzhou 510080, China; 4 Department of Endocrinology, the Third Affiliated Hospital of Sun Yat-sen University, Guangzhou 510630, China

**Keywords:** atherosclerosis, lipid rafts, NLRP3 inflammasome, NOX, pyroptosis

## Abstract

Lipid rafts play important roles in signal transduction, particularly in responses to inflammatory processes. The current study aimed to identify whether lipid raft-mediated inflammation contributes to hyperhomocysteinemia (HHcy)-accelerated atherosclerosis (AS), and to investigate the underlying mechanisms. THP-1-derived macrophages were used for in vitro experiments. ApoE^−/−^ mice were fed a high-fat diet for 12 weeks to establish an AS model, and a high-fat plus high-methionine diet was used to induce HHcy. We found that homocysteine (Hcy) increased the expression of p22^phox^ and p67^phox^ and promoted their recruitment into lipid rafts (indicating the assembly of the NOX complex), thereby increasing ROS generation and NOX activity, NLRP3 inflammasome activation, and pyroptosis. Mechanistically, Hcy activated the NOX-ROS-NLRP3 inflammasome pathway and induced pyroptosis by increasing the expression of acid sphingomyelinase (ASM) to promote the formation of lipid raft clustering. Importantly, lipid raft-mediated pyroptosis was confirmed in HHcy mice, and HHcy-promoted macrophage recruitment in atherosclerotic lesions and HHcy-aggravated AS were blocked by the lipid raft disruptor methyl-β-cyclodextrin. The study findings indicate that Hcy promotes lipid raft clustering via the upregulation of ASM, which mediates the assembly of the NOX complex, causing an increase in ROS generation, NLRP3 inflammasome activation, and pyroptosis, and contributes to HHcy-induced AS.

## 1. Introduction

Atherosclerosis (AS), a chronic inflammatory cardiovascular pathological phenomenon [1], is associated with high mortality worldwide. Homocysteine (Hcy) is an intermediate product in the metabolism of sulfur-containing amino acids [2]. An abnormal increase in total plasma Hcy results in a condition known as hyperhomocysteinemia (HHcy) [3], which is recognized as an independent risk factor for AS. HHcy induces the production of reactive oxygen species (ROS) and is associated with an increased vascular oxidative burden [4]. Additionally, oxidative stress has been proposed as a primary component of AS pathogenesis. ROS generated by nicotinamide adenine dinucleotide phosphate oxidase (NOX) was shown to serve as the primary source of ROS in cardiovascular systems [5]. Emerging evidence supports the facilitative effect of ROS overproduction on inflammation and AS [6]. Findings from previous studies have confirmed that HHcy induces inflammation and circulating monocyte differentiation through NOX-mediated oxidative stress [7]. Given that ROS is a fundamental component in the activation of the Nod-like receptor 3 (NLRP3) inflammasome [8], pyroptosis induced by the NLRP3 inflammasome is a potential programmed cell death process involved in the heightened inflammatory response induced by HHcy. Indeed, our previous study showed that HHcy mediated the activation of the NLRP3 inflammasome and promoted the release of IL-1β and IL-18 [9]. However, whether the NOX-induced production of ROS influences HHcy-aggravated AS is yet to be confirmed.

Macrophages play an integral role in the regulation of inflammation, and the pathophysiological processes induced by macrophages have been confirmed in the progression of AS [10]. Additionally, the proliferation, aggregation, and death of macrophages may promote the formation, enlargement, and rupture of lipid plaques [11]. Macrophages in atherosclerotic lesions are considered the primary source of proinflammatory cytokines [12]. Moreover, the activation of the NLRP3 inflammasome was also associated with macrophage recruitment into aortic lesions and plaque instability [13].

Lipid rafts are highly dynamic and low-density specialized microdomains of membranes enriched in sphingolipids, cholesterol, and special structural proteins [14]. Lipid rafts provide a platform for cell signaling and molecular complex assembly [15,16]. Receptors functioning in lipid rafts mediate cholesterol influx into macrophages and promote foam cell formation [17]. Reportedly, macrophage colony-stimulating factor stimulates lipid raft-associated NOX, inducing the generation of ROS, which influences the survival of macrophages [18]. Angiotensin II has been shown to promote the recruitment of NOX subunits into lipid rafts and the overproduction of ROS [19]. However, whether the NOX-induced activation of NLRP3 inflammasomes and pyroptosis via lipid rafts contributes to HHcy-induced AS remains elusive. Since inflammation is a key factor in AS, lipid rafts may promote the development of AS by participating in the regulation of inflammatory signaling pathways. Additionally, targeting of lipid rafts may be a potential strategy for the prevention and treatment of AS.

The present study aims to investigate whether lipid rafts mediate NLRP3 inflammasome activation and pyroptosis and contribute to HHcy-induced AS, and to determine the underlying mechanism.

## 2. Materials and Methods

### 2.1. Cell Culture

THP-1 cells were cultured in RPMI-1640 medium supplemented with 10% fetal bovine serum (FBS, Gibco, New York, NY, USA) and 100 U/mL penicillin and streptomycin in the presence of 5% CO_2_ at 37 °C. The differentiation of cells into macrophages was induced by treatment with 100 ng/mL phorbol-12-myristate-13-acetate (PMA, Sigma-Aldrich, St. Louis, MO, USA) for 24 h.

### 2.2. Small Interfering RNA (siRNA) Transfection

Macrophages were transfected with acid sphingomyelinase (ASM)-siRNA or control-siRNA (Santa Cruz Biotechnology, Santa Cruz, CA, USA) along with liposome Lipofectamine 3000 (Invitrogen, Waltham, MA, USA) according to the manufacturer’s instructions. Then, cells were transferred into a fresh culture medium and incubated with 100 µmol/L Hcy for an additional 24 h.

### 2.3. ROS Assessment

Intracellular and mitochondrial ROS levels were measured using dichloro-dihydro-fluorescein diacetate (DCFH-DA, Beyotime, Shanghai, China) and MitoSOX™ Red (Invitrogen) based on the manufacturer’s instructions. THP-1-derived macrophages were pretreated with 10 mmol/L methyl-β-cyclodextrin (MβCD, Sigma-Aldrich), lipid raft disruptor for 2 h, or with 10 µmol/L desipramine (DES, Sigma-Aldrich) for 15 min, and were then stimulated with 100 µmol/L Hcy (Sigma-Aldrich) for 24 h. Cells were loaded with DCFH-DA (10 μmol/L) for 30 min or MitoSOX™ Red (5 μmol/L) for 15 min at 37 °C in the dark. The fluorescence intensity was measured using DMI8 (Leica, Wetzlar, Germany) and Cytoflex S (Beckman Coulter, Brea, CA, USA).

### 2.4. Lipid Raft Extraction

Macrophages were pretreated with MβCD (10 mmol/L) for 2 h or DES (10 µmol/L) for 15 min and then treated with Hcy (100 µmol/L) for 24 h. The lipid raft and non-lipid raft fractions were extracted using the UltraRIPA kit (Funakoshi, Tokyo, Japan) in accordance with the manufacturer’s instructions.

### 2.5. NOX Activity Assessment

Since NOX catalyzes NADPH to NADP^+^, NOX activity can be determined using the NADP^+^/NADPH ratio [20]. Macrophages were pretreated with MβCD (10 mmol/L) for 2 h or DES (10 µmol/L) for 15 min and then treated with Hcy (100 µmol/L) for 24 h, and the cells were lysed and mixed with 200 μL of extraction buffer for NADP^+^/NADPH ratio detection using an assay kit (S0179, Beyotime) according to the manufacturer’s instructions.

### 2.6. Animal Model

Apolipoprotein E knockout (apoE^−/−^) mice (8-week-old male) (Vital River, Beijing, China) were housed in a controlled facility at approximately 24 ℃ under a 12 h light/dark cycle and provided free access to food and water. Mice were randomly divided into control, high-fat (HF), HHcy, and HHcy plus MβCD groups (*n* = 6 for each group). The control and HF mice were fed a regular diet and high-fat diet (HF, Dyets Inc, Wuxi, China) for 12 weeks, respectively. Mice in the HHcy and HHcy+MβCD groups were fed an HF plus high-methionine diet (D210305, Dyets Inc). MβCD was subcutaneously injected in the last 4 weeks of the treatment (2 g/kg, 2 times a week). At the end of the experiment, mice were sacrificed by exsanguination after anesthesia with the intraperitoneal injection of 80 mg/kg pentobarbital. Plasma and aorta samples were collected and stored at 80 °C for further analysis. All experiments were conducted in accordance with the Guide for the Care and Use of Laboratory Animals published by the US National Institutes of Health (No. 85–23, revised 1996). The research protocol was approved by the ethic committees of Sun Yat-sen University.

### 2.7. Histological Analysis of the Aortic Root Plaque

The aortic roots were isolated and embedded in an optimum cutting temperature compound and cut into 6 µm thick sections. Atherosclerotic lesions were evaluated by Oil Red O staining and imaged under the microscope (OLYMPUS BX51, Tokyo, Japan). The atherosclerotic lesion area was analyzed using the Image J software (NIH, Bethesda, MD, USA).

### 2.8. Enzyme-Linked Immunosorbent Assay (ELISA)

The plasma concentrations of Hcy and inflammatory cytokines (IL-1β and IL-18) were measured using ELISA kits (Cloud-Clone Corp, Katy, TX, USA; MultiSciences Biotech, Hangzhou, China) according to the manufacturer’s instructions. Intra-assay coefficients of variation (CVs), inter-assay CVs and limit of detection for IL-1β are 2.8–5.6%, 4.2–8.4% and 1.45 pg/mL, respectively. Intra-assay CVs, inter-assay CVs and limit of detection for IL-18 are 2.7–4.0%, 2.4–3.7 % and 0.93 pg/mL, respectively. Intra-assay CVs, inter-assay CVs and limit of detection for Hcy are <10%, 12% and 40.22 ng/mL, respectively.

### 2.9. Western Blot Analysis

Proteins were collected from macrophage lysates or mouse aorta. Western blot procedures were conducted as standard protocol with specific antibodies against P22^phox^ (1:1000, Santa Cruz Biotechnology), P67^phox^ (1:1000, Santa Cruz Biotechnology), ASM (1:1000, Santa Cruz Biotechnology), GSDMD (1:1000, Abcam, Cambridge, UK), Caspase-1 (1:1000, Abclonal, Wuhan, China), IL-1β (1:1000, ImmunoWay, Plano, TX, USA), NLRP3 (1:1000, Adipogen, San Diego, CA, USA), and β-actin (1:1000, Cell Signaling Technology, Danvers, MA, USA). The blots were visualized using an ImageQuant Las4000mini instrument (Cytiva, Marlborough, MA, USA). Western blot images were analyzed, and quantification was performed using the Image J software (NIH, Bethesda, MD, USA).

### 2.10. Immunofluorescence Analysis

Immunofluorescence staining was conducted as described previously [9]. The slides were subjected to double immunofluorescence staining for NLRP3 (1:100, Abcam) and F4-80 (1:100, Abcam) in the aortic root to verify the recruitment of macrophages. NLRP3 (1:100, Adipogen) and cleaved caspase-1 antibodies (1:100, ImmunoWay) were used to confirm the activation of the NLRP3 inflammasome. Cleaved caspase-1 (1:100, ImmunoWay) and TdT-mediated dUTP nick end labeling (TUNEL, Beyotime) were used to distinguish pyroptotic cells from apoptotic cells. Double staining with p22^phox^ or p67^phox^ (1:100, Santa Cruz Biotechnology) and CT-xB (1:100, Abcam, Cambridge, UK) was performed to verify the colocalization of NOX subunits with lipid rafts. Images were acquired using a laser scanning confocal microscope (LSM780, Zeiss, Jena, Germany).

### 2.11. Statistical Analysis

Data are presented as mean ± SD. All of the in vitro data were from at least three independent experiments. GraphPad Prism 9.0 software (GraphPad Software, Inc., San Diego, CA, USA) was used to analyze these results. Statistical comparisons between two groups were conducted using an unpaired Student’s *t*-test, and comparisons among multiple groups were performed using one-way ANOVA followed by the Least Significance Difference (LSD) test. Differences with *P* < 0.05 was considered statistically significant.

## 3. Results

### 3.1. MβCD Reduced the Intracellular and Mitochondrial ROS Levels in Hcy-Treated Macrophages

**Figure 1:**
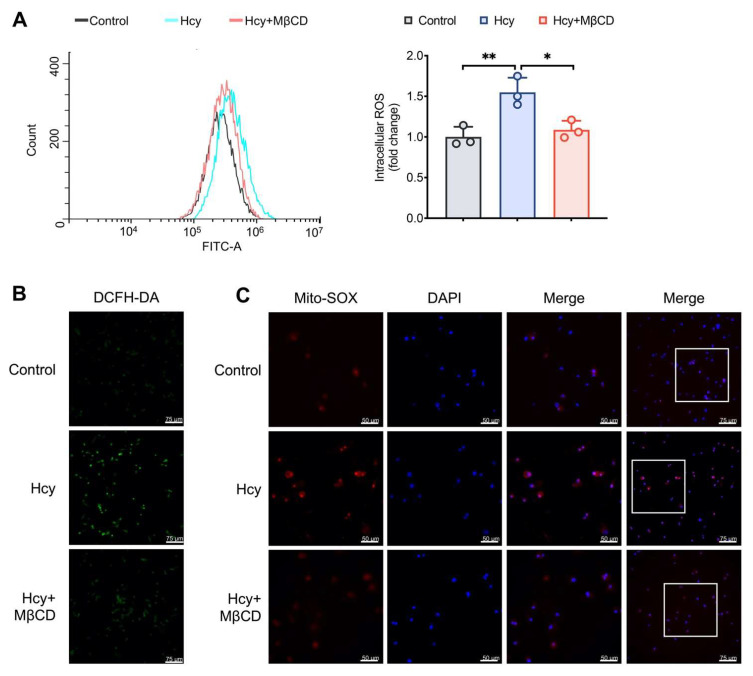
MβCD reduced both intracellular and mitochondrial ROS levels in Hcy-treated macrophages. (**A**) Representative flow cytometric image and quantitative analysis of the intracellular ROS levels. White squares: selected magnification area. (**B**,**C**) Representative confocal microscopic images of Mito-SOX and DCFH-DA. * *p* < 0.05, ** *p* < 0.01.

To determine whether lipid rafts contribute to the production of ROS, a cholesterol-depleting reagent MβCD was used to disrupt the integrity of lipid rafts. Hcy increased both the intracellular and mitochondrial ROS levels, which was reduced by the administration of MβCD (Figure 1A–C). These results indicated that the disruption of lipid rafts suppressed Hcy-induced ROS production.

### 3.2. MβCD Inhibited Hcy-induced p22^phox^ and p67^phox^ Recruitment into Lipid Rafts and Decreased NOX Activity in Macrophages

**Figure 2:**
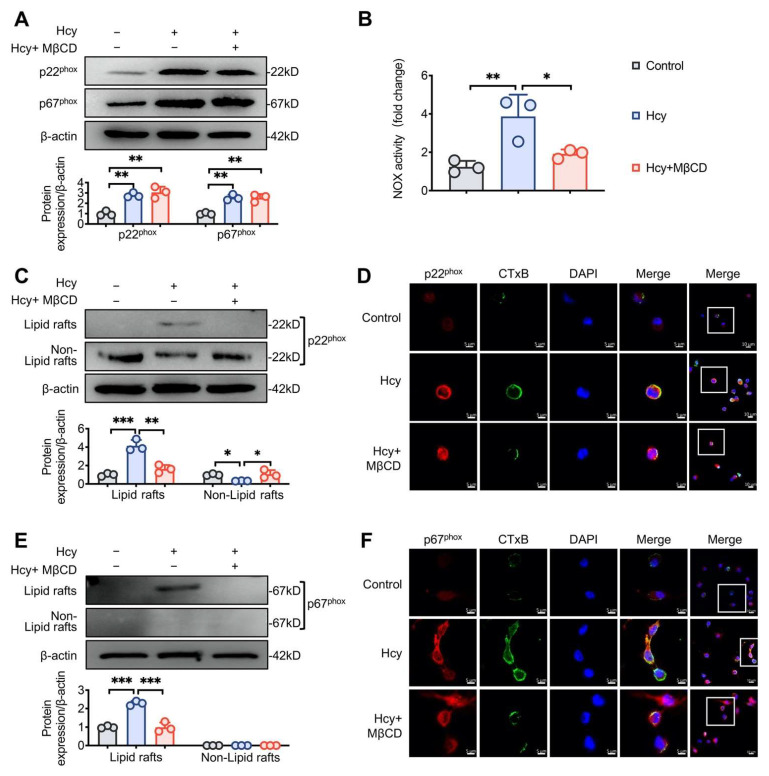
MβCD inhibited the Hcy-induced recruitment of p22^phox^ and p67^phox^ into lipid rafts and decreased NOX activity in macrophages. (**A**,**C**,**E**) Representative immunoblots and the corresponding quantification of the indicated proteins and (**B**) NOX activity in macrophages. (**D,F**) Representative confocal microscopic images of p22^phox^/p67^phox^ and CTxB. White squares: selected magnification area. * *p* < 0.05, ** *p* < 0.01 and *** *p* < 0.001.

NOX is one of the key sources of ROS production in cardiovascular systems. NOX-related oxidative stress serves as a key component in AS development. We found that Hcy significantly increased the expression of the two NOX subunits, p22^phox^ and p67^phox^, whereas MβCD did not influence the Hcy-induced upregulation of p22^phox^ and p67^phox^ (Figure 2A). However, Hcy-induced NOX activity was reduced by MβCD (Figure 2B). To further determine the underlying mechanism, the lipid raft and non-lipid raft fractions were extracted to assess the recruitment of p22^phox^ and p67^phox^ in lipid rafts. Hcy promoted the recruitment of p22^phox^ and p67^phox^ into the lipid raft domain, and MβCD prevented the aggregation of NOX subunits into lipid rafts (Figure 2C,E). Moreover, immunofluorescence staining for CTxB, a marker for lipid rafts, was performed to confirm lipid raft clustering. After Hcy treatment, weak fluorescence in a diffused punctuate staining pattern transformed to intensive fluorescence in a continuous-patch staining pattern, suggesting that Hcy promoted lipid raft clustering. We also confirmed that Hcy promoted the recruitment of p22^phox^ and p67^phox^ into lipid rafts, as evidenced by the increased colocalization of CTxB with p22^phox^ and p67^phox^, respectively (Figure 2D,F). These findings indicated that Hcy induces the assembly of the NOX complex in lipid rafts, thereby enhancing ROS generation.

### 3.3. MβCD Inhibited Hcy-Induced NLRP3 Inflammasome Activation and Pyroptosis in Macrophages

**Figure 3:**
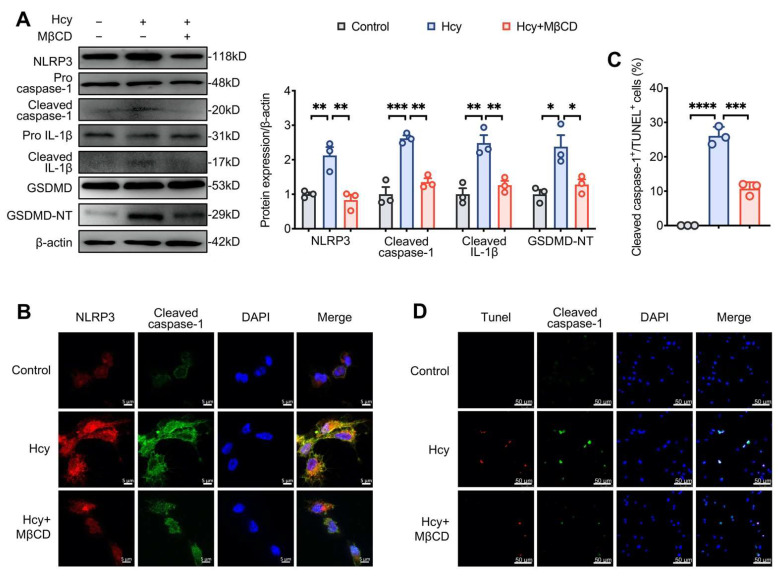
MβCD inhibited Hcy-induced NLRP3 inflammasome activation and pyroptosis in macrophages. (**A**) Representative blots and quantitative analysis of the expression of NLRP3, cleaved caspase-1, IL-1β and GSDMD. (**B**) Representative confocal microscopic images showing the colocalization of NLRP3 (red) with cleaved caspase-1 (green). (**C**,**D**) Representative images showing the colocalization of TUNEL (red) staining with cleaved caspase-1 (green) and the percentage of cleaved caspase-1^+^/TUNEL^+^ cells (pyroptotic cells). * *p* < 0.05, ** *p* < 0.01, *** *p* < 0.001, and **** *p* < 0.0001.

MβCD suppressed the expression of NLRP3 and the cleavage of caspase-1, IL-1β, and GSDMD in Hcy-treated macrophages (Figure 3A), indicating that it suppressed Hcy-induced NLRP3 inflammasome activation and pyroptosis. To further confirm the results, we assessed the co-localization of NLRP3 and cleaved caspase-1 by immunofluorescence analysis. The Hcy-induced colocalization of NLRP3 and cleaved caspase-1 was inhibited by MβCD (Figure 3B). Moreover, pyroptosis is characterized by DNA fragmentation, membrane pore formation and proinflammatory content release [21], and DNA fragmentation can be observed by TUNEL staining. Cleaved caspase 1^+^/TUNEL^+^ cells were defined as pyroptotic cells. Additionally, MβCD reduced the pyroptotic index in Hcy-treated cells (Figure 3C,D).

### 3.4. Disruption of Lipid Raft Clustering Suppressed Hcy-Induced NLRP3 Inflammasome Activation and Pyroptosis in Macrophages

**Figure 4:**
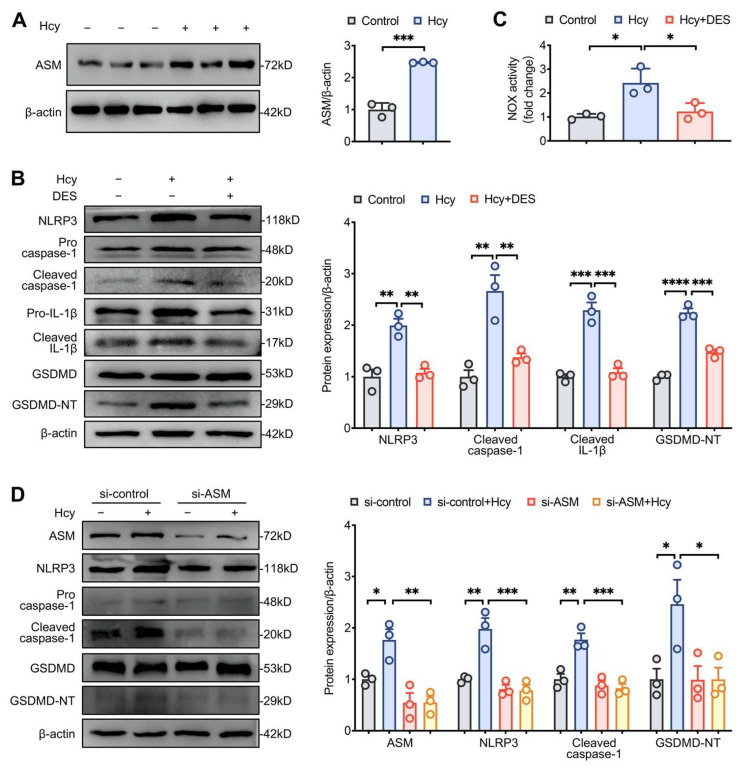
Disruption of lipid raft clustering suppressed Hcy-induced NLRP3 inflammasome activation and pyroptosis in macrophages. (**A**) Representative blots and quantitative analysis results showing the expression of ASM. (**B**,**D**) Representative blots and quantitative analysis results showing the expression of NLRP3, cleaved caspase-1, IL-1β and GSDMD. (**C**) NOX activity in macrophages. * *p* < 0.05, ** *p* < 0.01, *** *p* < 0.001 and **** *p* < 0.0001.

ASM hydrolyzes membrane lipid sphingomyelin to ceramide (Cer), which initiates and facilitates the clustering of lipid rafts in the cell membrane [22]. Hcy increased the expression of ASM and NLRP3 and promoted the cleavage of caspase-1, IL-1β, and GSDMD. Importantly, the ASM-specific inhibitor DES inhibited Hcy-induced NLRP3 inflammasome activation and pyroptosis as well as NOX activity (Figure 4A–C). To further confirm the role of lipid raft clustering, the results of gene-silencing by ASM-siRNA were confirmed by Western blotting analysis. Compared to the results obtained using the control-siRNA, ASM silencing significantly suppressed the expression of NLRP3 and the cleavage of caspase-1 and GSDMD induced by Hcy (Figure 4D). These data indicate that Hcy induced NLRP3 inflammasome activation and pyroptosis by enhancing the expression of ASM to promote lipid raft clustering.

### 3.5. MβCD Decreased the Levels of Plasma Inflammatory Mediators and Attenuated Atherosclerosis in HHcy Mice

**Figure 5:**
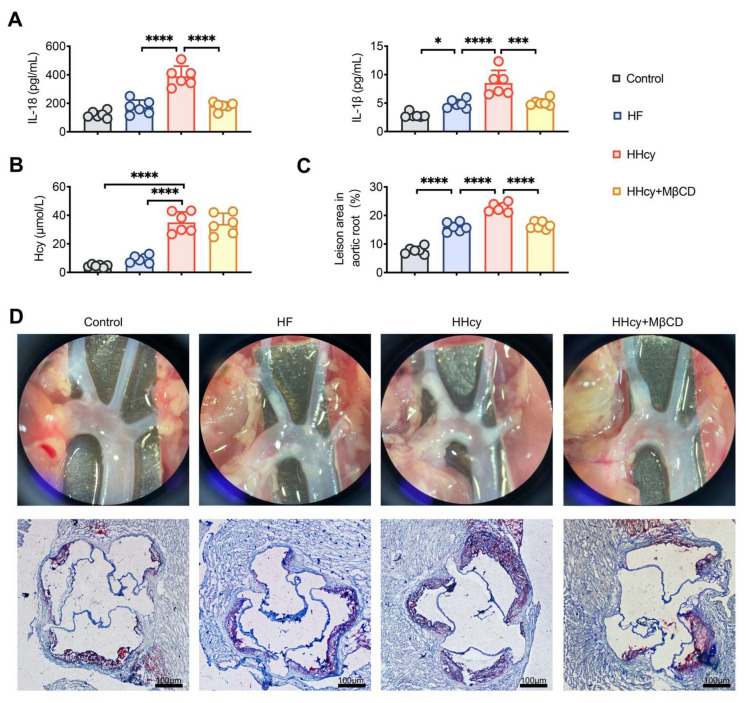
MβCD decreased the levels of plasma inflammatory mediators and attenuated atherosclerosis in HHcy-induced mice. Plasma levels of IL-1β and IL-18 (**A**) and Hcy (**B**). (**C**,**D**) Oil red O staining showing the atherosclerotic lesions, and quantitative analysis of the atherosclerotic lesion area in the aortic root. *n* = 6 for each group. * *p* < 0.05, *** *p* < 0.001 and **** *p* < 0.0001.

Our previous findings confirmed that the activation of NLRP3 inflammasomes plays an essential role in HHcy-induced AS [9]. Here, we conducted further investigations to determine whether lipid rafts play a role in HHcy-accelerated AS in apoE^−/−^ mice. Compared to HF mice, HHcy-induced mice showed higher plasma levels of the proinflammatory cytokines IL-1β and IL-18 (Figure 5A). Similarly, Oil Red O staining showed that the plaque area of the aortic root was significantly greater in HHcy mice, and treatment with MβCD significantly decreased the plasma levels of IL-1β and IL-18 and the atherosclerotic plaque area in HHcy mice (Figure 5A,C,D). Moreover, compared to that in HF mice, the plasma Hcy levels increased considerably in HHcy mice. MβCD did not affect the level of plasma Hcy (Figure 5B), suggesting that MβCD exerted an anti-atherosclerotic effect by inhibiting inflammation but not decreasing the plasma level of Hcy. These results demonstrated that MβCD suppresses HHcy-induced inflammation and inhibits HHcy-induced atherosclerosis in apoE^−/−^ mice.

### 3.6. MβCD Inhibited HHcy-Induced NLRP3 Inflammasome Activation, Pyroptosis, and Macrophage Recruitment into the Aortic Root Plaque in ApoE^−/−^ Mice

**Figure 6:**
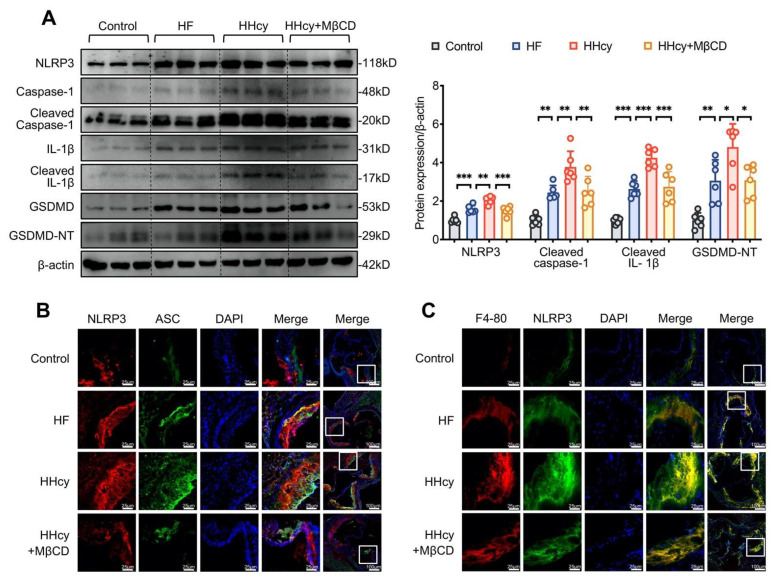
MβCD inhibited HHcy-induced NLRP3 inflammasome activation, pyroptosis, and macrophage recruitment into the aortic root plaque in apoE^−/−^ mice. (**A**) Representative blots and quantitative analysis, showing the expression of NLRP3, cleaved caspase-1, IL-1β, and GSDMD in the aorta. (**B,C**) Representative confocal microscopic images, showing the colocalization of NLRP3 (red) with ASC (green) and that of F4-80 (red) with NLRP3 (green). White squares: selected magnification area. *n* = 6 for each group, * *p* < 0.05, ** *p* < 0.01, *** *p* < 0.001.

HHcy induced NLRP3 inflammasome activation, as confirmed by the increased expression of NLRP3, cleaved caspase-1, IL-1β, and GSDMD, as well as the colocalization of NLRP3 inflammasome components (NLRP3 and cleaved caspase-1) detected by Western blotting and confocal microscopy, respectively. Consistent with our in vitro results, HHcy-induced NLRP3 inflammasome activation and pyroptosis was reversed by MβCD, indicating that lipid rafts play a vital role in the progression of HHcy-induced AS (Figure 6A,B). The recruitment and death of macrophages contribute to the formation and enlargement of AS plaques [11]. Double immunofluorescent staining with anti-NLRP3 and -F4-80 (macrophage marker) antibodies was performed to explore the recruitment of macrophages. In atherosclerotic lesions, NLRP3 and F4-80 showed marginal co-localization in control mice and relatively greater co-localization in HF mice, whereas they largely showed enhancement and colocalization in HHcy mice. After MβCD intervention, the fluorescence intensity of NLRP3 and F4-80 was weakened, and less co-localization was observed, indicating that lipid rafts are involved in HHcy-induced macrophage infiltration in the atherosclerotic lesion (Figure 6C).

## 4. Discussion

The present study is the first to provide evidence that HHcy promotes AS through lipid raft-mediated pyroptosis. Mechanistically, Hcy induces p22^phox^ and p67^phox^ recruitment into lipid rafts, leading to NOX complex assembly and ROS generation, which consequently activates the NLRP3 inflammasome and induces pyroptosis. Moreover, the destruction of lipid rafts or the silencing of ASM for interference with lipid raft clustering successfully suppressed the pro-pyroptotic effect of Hcy via inhibition of the NOX-ROS-NLRP3 pathway. Our results suggest that the targeting of lipid rafts to blunt HHcy-induced NLRP3 inflammasomes and pyroptosis could exert an atheroprotective effect.

HHcy, an independent cardiovascular risk factor, is associated with vascular inflammation and AS. HHcy enhances the adhesion between monocytes and endothelial cells and promotes monocyte differentiation, macrophage maturation, and uptake of ox-LDL by macrophages. HHcy-induced oxidative stress has been confirmed to mediate endothelial dysfunction [23]. Our previous study showed that Hcy promotes ROS generation and enhances vascular inflammation, which aggravates the progression of AS [9]. However, the mechanism underlying Hcy-induced ROS generation remains unknown. NOX is considered one of the main resources for ROS production in endothelial cells. NOX is a multi-subunit complex, composed of the cytosolic components of regulatory subunits (p47phox, p67phox, p40phox, and small G-protein Rac1/2) and the membrane components of catalytic subunits (gp91phox and p22phox); the cytoplasmic subunits are translocated to the membrane and bind to the membrane subunits to assemble the active form of the NOX complex in response to stimulus [24]. Excessive assembly and activation of the NOX complex results in the induction of chronic inflammatory diseases, such as AS [18]. Reportedly, Hcy-stimulated NOX-dependent oxidative stress results in endothelial dysfunction and induces the vascular inflammatory response [25,26]. H_2_O_2_ generated by NOX is required to promote mitochondrial ROS production [27].

Lipid rafts are considered an essential platform for the aggregation and assembly of NOX subunits, and thus, form a redox signaling platform to produce ROS and conduct redox signaling. Lipid rafts are small dynamic cholesterol- and sphingolipid-enriched microdomains [28]. Compared to the surrounding membrane, lipid rafts exhibit a more ordered and tightly packed structure. Lipid rafts are involved in various biological events, such as protein transport and apoptosis, because they provide a stable platform and contribute to the mediation and amplification of various cellular signals [29,30]. In the present study, MβCD was administered to disrupt intact lipid rafts. Correspondingly, we confirmed that Hcy promoted the production of ROS, which was inhibited by MβCD. Observation of the colocalization of lipid rafts and NOX subunits via confocal microscopy is a widely accepted method for the detection of NOX subunit recruitment in lipid rafts [16]. In our study, we identified that Hcy increased the expression of p22^phox^ and p67^phox^ and substantially enhanced NOX activity, which was accompanied by increased intracellular and mitochondrial ROS generation in macrophages. Furthermore, we observed that Hcy triggered p22^phox^ and p67^phox^ aggregation and translocation, respectively, into lipid raft fractions. Meanwhile, Hcy enhanced the enrichment of p22^phox^ and p67^phox^ in lipid rafts, as evidenced by the increased colocalization of CTxB and p22^phox^ or p67^phox^. Taken together, our results demonstrated, for the first time, that Hcy induces ROS generation via the assembly of the NOX complex in lipid rafts.

It is widely accepted that chronic progressive inflammation has deleterious and additive effects that accelerate AS and contribute to lesion rupture [31]. HHcy promotes pro-inflammatory cytokine production and lipid accumulation in macrophages and has been regarded as a major driving force in the development of atherosclerotic plaques. Pyroptosis is a form of programmed cell death, and NLRP3 inflammasome-related pyroptosis is characterized by caspase-1-dependent pore formation and pro-inflammatory factor (such as IL-1β and IL-18) release, leading to cell lysis. Previous research has shown that cholesterol crystals could trigger NLRP3 inflammasome activation, and that excessive caspase-1 expression is associated with the instability of the atherosclerotic plaque [32]. Frank et al. reported that the deficiency of caveolin-1, a lipid raft resident protein, is associated with a protective effect against AS [33]. Notably, our results showed that MβCD reduced the atherosclerotic area in HHcy mice, suggesting that lipid rafts play a vital role in HHcy-induced AS.

Lipid rafts play an essential role in many cellular function regulations, particularly in inflammation [34]. Previous studies have demonstrated that lipid rafts provide a stable platform for the assembly of the IFNR complex and are involved in IFN-stimulated inflammatory signaling [35]. Reportedly, the disruption of lipid rafts inhibits the recruitment of Toll-like receptor 4 (TLR4) and myeloid differentiation factor 88 (MyD88) into lipid rafts, and alleviates lipopolysaccharide-mediated inflammation in macrophages [36]. Consistent with the findings from our previous study [13], the activation of the NLRP3 inflammasome could be prevented by destroying the integrity of lipid rafts. Moreover, MβCD significantly decreased the levels of pro-inflammatory factors in the peripheral blood of HHcy mice. Pyroptosis is characterized by the formation of plasma membrane pores, the release of pro-inflammatory cytokines, and cell lysis, and could be triggered by NLRP3 inflammasome activation [37]. Most studies have focused on the pro-apoptotic properties of lipid rafts in various diseases. A novel finding of this study is that we confirmed that lipid rafts are associated with HHcy-induced pyroptosis, and that the destruction of lipid rafts helps to efficiently attenuate pyroptosis as well as consequent atherosclerotic lesion formation in apoE^−/−^ mice.

ASM, a major member of sphingomyelinases, can be translocated into the cell membrane to hydrolyze sphingomyelin to Cer [38,39]. Cer facilitates the combination of individual lipid rafts into larger and stable Cer-enriched membrane microdomains in response to different stimuli, further enhancing protein interactions and amplifying signal transduction [13,40]. Consistent with this, we found that Hcy increased the expression of ASM. DES, an ASM inhibitor, interrupted lipid raft clustering and inhibits the assembly of the NOX complex, as evidenced by the decreased activity of NOX. To further confirm the role of lipid raft clustering in Hcy-mediated inflammation, we induced siRNA-mediated ASM knockdown to interrupt lipid raft clustering. Our results demonstrated that, consistent with the results of treatment with DES, ASM silencing abrogated NLRP3 inflammasome activation and pyroptosis in Hcy-treated macrophages. It was reported that ASM induced by cigarette smoke markedly increases extracellular vesicle (EVs) production [41]. Recent study highlighted that monocytic-derived EVs encapsulate caspase-1 and promote macrophages apoptosis [42]. Our findings are the first to show that the inhibition of lipid raft clustering exerts a protective effect on Hcy-induced NLRP3 inflammasome activation and pyroptosis in macrophages. However, it is unclear whether EVs are involved in NLRP3 inflammasome activation and pyroptosis in HHcy model. Expanding studies are warranted to explore the effect of ASM-mediated lipid raft clustering in EVs production and dissect its role in NOX-ROS-NLRP3 activation and pyroptosis in HHcy-induced AS in vivo.

## 5. Conclusion

**Figure 7:**
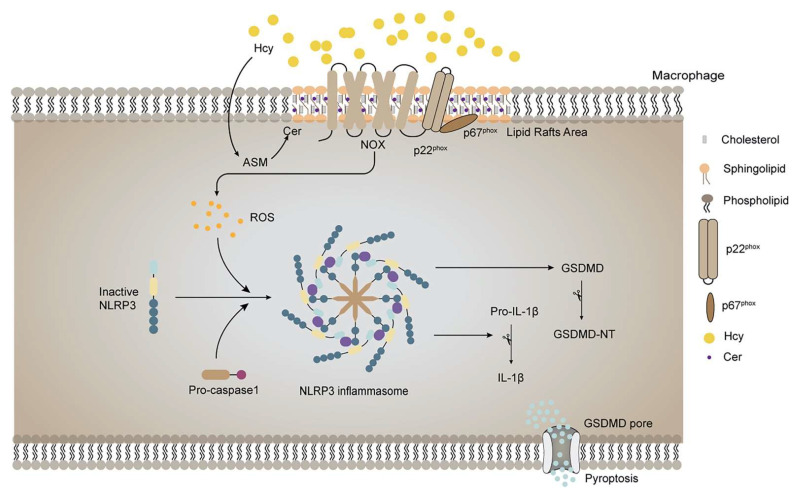
Graphic abstract. HHcy induces pyroptosis and atherosclerosis via the lipid raft-mediated NOX-ROS-NLRP3 inflammasome pathway.

Taken together, this is the first study to demonstrate that Hcy promotes the assembly of the NOX complex through ASM-mediated lipid raft clustering, which increases ROS generation, NLRP3 inflammasome activation, and pyroptosis, eventually contributing to the progression of AS (Figure 7). The NOX-ROS-NLRP3 inflammasome pathway may be a potential therapeutic target against HHcy-induced pyroptosis and AS.

## Data Availability

Data are available upon reasonable request from the corresponding author.

## References

[1] Zhuang T., Liu J., Chen X., Zhang L., Pi J., Sun H., Li L., Bauer R., Wang H., Yu Z. (2019). Endothelial Foxp1 Suppresses Atherosclerosis via Modulation of Nlrp3 Inflammasome Activation. Circ. Res..

[2] Guo W., Zhang H., Yang A., Ma P., Sun L., Deng M., Mao C., Xiong J., Sun J., Wang N. (2020). Homocysteine accelerates atherosclerosis by inhibiting scavenger receptor class B member1 via DNMT3b/SP1 pathway. J. Mol. Cell. Cardiol..

[3] Cruciani-Guglielmacci C., Meneyrol K., Denom J., Kassis N., Rachdi L., Makaci F., Migrenne-Li S., Daubigney F., Georgiadou E., Denis R. (2022). Homocysteine Metabolism Pathway Is Involved in the Control of Glucose Homeostasis: A Cystathionine Beta Synthase Deficiency Study in Mouse. Cells.

[4] Antoniades C., Antonopoulos A., Tousoulis D., Marinou K., Stefanadis C. (2009). Homocysteine and coronary atherosclerosis: From folate fortification to the recent clinical trials. Eur. Heart J..

[5] Sahoo S., Meijles D., Pagano P. (2016). NADPH oxidases: Key modulators in aging and age-related cardiovascular diseases?. Clin. Sci..

[6] Poznyak A.V., Grechko A.V., Orekhova V.A., Khotina V., Ivanova E.A., Orekhov A.N. (2020). NADPH Oxidases and Their Role in Atherosclerosis. Biomedicines.

[7] Zhang M., Hou Y., Shen Y., Guo X., Shang D., Zhang D. (2018). Probucol reverses homocysteine induced inflammatory monocytes differentiation and oxidative stress. Eur. J. Pharmacol..

[8] Tschopp J., Schroder K. (2010). NLRP3 inflammasome activation: The convergence of multiple signalling pathways on ROS production?. Nat. Rev. Immunol..

[9] Wang R., Wang Y., Mu N., Lou X., Li W., Chen Y., Fan D., Tan H. (2017). Activation of NLRP3 inflammasomes contributes to hyperhomocysteinemia-aggravated inflammation and atherosclerosis in apoE-deficient mice. Lab. Investig..

[10] Chen W., Schilperoort M., Cao Y., Shi J., Tabas I., Tao W. (2022). Macrophage-targeted nanomedicine for the diagnosis and treatment of atherosclerosis. Nat. Rev. Cardiol..

[11] Martinet W., Coornaert I., Puylaert P., De Meyer G. (2019). Macrophage Death as a Pharmacological Target in Atherosclerosis. Front. Pharmacol..

[12] Zeng W., Wu D., Sun Y., Suo Y., Yu Q., Zeng M., Gao Q., Yu B., Jiang X., Wang Y. (2021). The selective NLRP3 inhibitor MCC950 hinders atherosclerosis development by attenuating inflammation and pyroptosis in macrophages. Sci. Rep..

[13] Duan F., Zeng C., Liu S., Gong J., Hu J., Li H., Tan H. (2021). α1-nAchR-Mediated Signaling Through Lipid Raft Is Required for Nicotine-Induced NLRP3 Inflammasome Activation and Nicotine-Accelerated Atherosclerosis. Front. Cell Dev. Biol..

[14] Regen S. (2020). The Origin of Lipid Rafts. Biochemistry.

[15] Jin S., Zhou F. (2009). Lipid raft redox signaling platforms in vascular dysfunction: Features and mechanisms. Curr. Atheroscler. Rep..

[16] Li P., Zhang Y., Yi F. (2007). Lipid raft redox signaling platforms in endothelial dysfunction. Antioxid. Redox Signal..

[17] Li Q., Liu X., Zhang X., Du Y., Chen G., Xiang P., Ling W., Wang D. (2022). Terpene Lactucopicrin Limits Macrophage Foam Cell Formation by a Reduction of Lectin-Like Oxidized Low-Density Lipoprotein Receptor-1 in Lipid Rafts. Mol. Nutr. Food Res..

[18] Schmitz G., Grandl M. (2007). Role of redox regulation and lipid rafts in macrophages during Ox-LDL-mediated foam cell formation. Antioxid. Redox Signal..

[19] Gu M., Fu Y., Sun X., Ding Y., Li C., Pang W., Pan S., Zhu Y. (2012). Proteomic analysis of endothelial lipid rafts reveals a novel role of statins in antioxidation. J. Proteome Res..

[20] Wen Y., Chen H., Zhang L., Wu M., Zhang F., Yang D., Shen J., Chen J. (2021). Glycyrrhetinic acid induces oxidative/nitrative stress and drives ferroptosis through activating NADPH oxidases and iNOS, and depriving glutathione in triple-negative breast cancer cells. Free Radic. Biol. Med..

[21] He X., Fan X., Bai B., Lu N., Zhang S., Zhang L. (2021). Pyroptosis is a critical immune-inflammatory response involved in atherosclerosis. Pharmacol. Res..

[22] Zhang A., Yi F., Jin S., Xia M., Chen Q., Gulbins E., Li P. (2007). Acid sphingomyelinase and its redox amplification in formation of lipid raft redox signaling platforms in endothelial cells. Antioxid. Redox Signal..

[23] Lin C., Chen Y., Leu H., Lin S., Chen Y., Huang S., Chen J. (2009). Anti-inflammatory strategies for homocysteine-related cardiovascular disease. Front. Biosci.-Landmark.

[24] Elumalai S., Karunakaran U., Moon J.-S., Won K.-C. (2021). NADPH Oxidase (NOX) Targeting in Diabetes: A Special Emphasis on Pancreatic β-Cell Dysfunction. Cells.

[25] Edirimanne V., Woo C., Siow Y., Pierce G., Xie J., O K. (2007). Homocysteine stimulates NADPH oxidase-mediated superoxide production leading to endothelial dysfunction in rats. Can. J. Physiol. Pharmacol..

[26] Li Y., Zhao Q., Cao Y., Si J., Li J., Cao K., Pang X. (2021). Probucol decreases homocysteine-stimulated CRP production in rat aortic smooth muscle cells via regulating HO-1/NADPH oxidase/ROS/p38 pathway. Acta Biochim. Biophys. Sin..

[27] Ahmed K., Sawa T., Ihara H., Kasamatsu S., Yoshitake J., Rahaman M., Okamoto T., Fujii S., Akaike T. (2012). Regulation by mitochondrial superoxide and NADPH oxidase of cellular formation of nitrated cyclic GMP: Potential implications for ROS signalling. Biochem. J..

[28] Lingwood D., Simons K. (2010). Lipid rafts as a membrane-organizing principle. Science.

[29] Li B., Qin Y., Yu X., Xu X., Yu W. (2022). Lipid raft involvement in signal transduction in cancer cell survival, cell death and metastasis. Cell Prolif..

[30] Wang S., Yuan S., Peng D., Zhao S. (2010). High-density lipoprotein affects antigen presentation by interfering with lipid raft: A promising anti-atherogenic strategy. Clin. Exp. Immunol..

[31] Xiong J., Ma F., Ding N., Xu L., Ma S., Yang A., Hao Y., Zhang H., Jiang Y. (2021). miR-195-3p alleviates homocysteine-mediated atherosclerosis by targeting IL-31 through its epigenetics modifications. Aging cell.

[32] Xu Y., Zheng L., Hu Y., Wang Q. (2018). Pyroptosis and its relationship to atherosclerosis. Clin. Chim. Acta.

[33] Frank P., Lee H., Park D., Tandon N., Scherer P., Lisanti M. (2004). Genetic ablation of caveolin-1 confers protection against atherosclerosis. Arterioscler. Thromb. Vasc. Biol..

[34] Sun M., Han X., Zhou D., Zhong J., Liu L., Wang Y., Ni J., Shen X., Liang C., Fang H. (2021). BIG1 mediates sepsis-induced lung injury by modulating lipid raft-dependent macrophage inflammatory responses. Acta Biochim. Biophys. Sin..

[35] Lee J., Han J., Woo J., Jou I. (2022). 25-Hydroxycholesterol suppress IFN-γ-induced inflammation in microglia by disrupting lipid raft formation and caveolin-mediated signaling endosomes. Free Radic. Biol. Med..

[36] Chen J., Ullah H., Zheng Z., Gu X., Su C., Xiao L., Wu X., Xiong F., Li Q., Zha L. (2020). Soyasaponins reduce inflammation by downregulating MyD88 expression and suppressing the recruitments of TLR4 and MyD88 into lipid rafts. BMC Complementary Med. Ther..

[37] Song Z., Gong Q., Guo J. (2021). Pyroptosis: Mechanisms and Links with Fibrosis. Cells.

[38] Jia S., Jin S., Zhang F., Yi F., Dewey W., Li P. (2008). Formation and function of ceramide-enriched membrane platforms with CD38 during M1-receptor stimulation in bovine coronary arterial myocytes. Am. J. Physiol. Heart Circ. Physiol..

[39] Silva L., Futerman A., Prieto M. (2009). Lipid raft composition modulates sphingomyelinase activity and ceramide-induced membrane physical alterations. Biophys. J..

[40] Megha, London E. (2004). Ceramide selectively displaces cholesterol from ordered lipid domains (rafts): Implications for lipid raft structure and function. J. Biol. Chem..

[41] Serban K., Rezania S., Petrusca D., Poirier C., Cao D., Justice M., Patel M., Tsvetkova I., Kamocki K., Mikosz A. (2016). Structural and functional characterization of endothelial microparticles released by cigarette smoke. Sci. Rep..

[42] Charla E., Mercer J., Maffia P., Nicklin S.A. (2020). Extracellular vesicle signalling in atherosclerosis. Cell Signal.

